# An Examination of Dr. Buxton's Mode of Treating Consumption, with Arguments against the Efficacy of High Temperature in That Disease

**Published:** 1815-03

**Authors:** Thomas Sutton

**Affiliations:** Croom's Hill


*For the Medical and Physical Journal.*

### *An Examination of Dr. Buxton's Mode of Treating Consumption, with Arguments against the Efficacy of High Temperature in that Disease;* by T. SUTTON, M.D.

I FIND several communications have been lately made by Dr. Buxton to the Monthly Magazine, for the purpose of supporting his theory of the efficacy of high and equable temperature in consumption; and I also perceive a criticism in the Eclectic Review on Dr. Southey's work on Consumption, in which the writer avowedly advocates the same cause. I propose to make use of the materials supplied by these authors, to show the actual ground on which they stand by the statement they have made respecting the prevalence of consumption in various climates; and I am happy to use these materials, because they stand approved by the parties alluded to and myself.

The creed of Dr. Buxton and his friend the critic of the article in the Eclectic Review, respecting the cure of consumption by a high and equable temperature, may be readily stated in the doctor's own words from the Monthly Magazine. "A regular high temperature should be persevered in during the winter, on the supposition that these complaints (consumption, &c.) are chiefly produced and continued by variable and severe weather." "A most important remedy to oppose their progress (consumption, &c.) is warmth of temperature." "I conceived that there could scarcely be a possibility of its (the propriety of the application of such temperature in consumption) being disputed." The doctor has, however, found it to be disputed by me, and the critic complains of the opposition that the doctor's plan had encountered, and commends his zeal in striving against this opposition. Hence the doctor has made an appeal through the Monthly Magazine, and the critic has endeavoured to say all he can in support of his friend's doctrines, and, of course, little in favour of my sentiments on the same subject. It is now, therefore, incumbent upon me to follow them in the course course they have thought proper to pursue, and to make a free use of their own materials, for the purpose of avoiding the possibility of our disagreeing about the most important facts on which they are disposed to rest their discussions in the present instance.

I shall develope these facts in my own way, which will, I hope, be found to be clear and evident, and arranged according to the natural order of the subject; and I shall begin by taking it for granted, which those who may hold a contrary opinion of the treatment of the disease will readily admit, that consumption commences, and prevails most, in the severe season of the year, in this country. Having admitted this, the first inquiry, according to our present plan, will naturally be to investigate in how far severe weather tends to create it, by a reference to the accounts of the prevalence of this disease in cold climates. On turning to the critic's facts as alluded to, he states that the inhabitants of Vienna and some parts of Germany are very subject to consumption, while other countries, in a more northern latitude than we inhabit, enjoy a comparative freedom from the disease, for instance Denmark, Sweden, Russia, and Holland. The Dutch, who in other respects inhabit a sickly climate, are proverbially free from consumption. Among the diseases of Canada, consumption is not mentioned. With these facts before our eyes, how can we infer generally, that severe weather causes it, and that cold is to be avoided under its attacks as the greatest evil—when we view the number and magnitude of the countries in severe climates, which escape the scourge of this disease. Are the people of Vienna, &c. more negligent of their health than their other northern neighbours; and are they also inattentive to their clothing, their buildings, &c.? Why does Canada enjoy an immunity from consumption? inhabited by a people mostly sprung from among us and from France, in both which countries consumption prevails, and carrying also with them, as far as the climate will allow, their European customs, habits, and amusements. From these facts, then, I infer that severe weather cannot be the actual and chief cause of consumption, because, if it were, we might even expect the prevalence of this disorder according to the relative severity of temperature in different climates, and a sort of ratio of disease as we verge gradually into the warmer countries, which will appear not to be the case. The proposition, therefore, cannot be maintained on these grounds, which states variable and severe weather to be the chief cause of consumption. This evidence does not, however, invalidate part of the doctor's conclusion, that the disease may be carried (to a

 certain certain extent) by variable weather. While this is maintained to be a fact, we see the reason of consumption prevailing in this country in a greater degree in the colder seasons, because the temperature then varies more than in the other parts of the year. But it appears necessary to the plan of those who support the equable and high temperature treatment, to give a great part of the blame of exciting consumption to the severe weather, because they thence infer that a continued high temperature, in opposition to their supposed exciting cause, must be a proper remedy.

Let us now see what are related to be the effects of heat of climate in relation to consumption. I willingly follow these authorities for the information. Next to our own country, France and the south of Europe seem to be the regions most visited by this disease. Madeira is particularly so, and the disorder prevails much in Sicily. Do these facts afford us any useful analogy on which we can ground a practice of using high temperature, in imitation of the climate of southern countries, for the cure of consumption? If, in reality, we adopt any mode of temperature from observations grounded on the prevalence or exemption from consumption, in the various climates of Europe, we must endeavour to imitate a Dutch, a Danish, a Swedish, or Russian temperature.

Dr. Buxton has also travelled into tropical climates for information on this subject, and finds, by referring to authors, that consumption does not prevail in those latitudes so generally as with us. He is, therefore, led to conclude, and to rest his defence of equable high temperature for the cure of this disease, on what is related respecting its occurrence in those hot climates; but in these researches he does not find that perfect coincidence he appears to wish. Thus, respecting Jamaica, the most clear and authentic account on this subject contradicts the vague representations of others, and the doctor is pleased to find as a solution of the cause of more consumption being found to prevail in that island, that it varies more in the degrees of temperature than any other in the West Indies. He makes the like remark respecting Sicily. But does he recollect that this can serve him nothing in regard to his ground-work proposition, in which he asserts the origin of consumption to be derived chiefly from severe and variable weather, while in these cases, however he may rely on the variable, he must relinquish the very corner-stone of his edifice for the support of his high equable temperature, for the cure of consumption, by allowing this disease to exist in countries where the term *severe weather* cannot be applied. This opinion is also strongly opposed by the very authentic evidence that the disease prevails least of all in some of the most severe climates of Europe, and in the severe colds of Canada.

In drawing an inference in favour of the climates of the southern countries of Europe for the cure of consumption, it must be evident, that there has not existed a most perfect information on the subject. But, on a supposition that the disease does not prevail so much in some countries as in others, does it prove absolutely the propriety of sending such patients into those climates. I think decidedly not. There is another and a more important fact in the inquiry to be ascertained, and which appears to have been somewhat neglected: it is this, whether, when the disease actually occurs, it is more or less fatal in any proposed climate than in that in which the patient lives. I consider it to be of no consequence to contend whether there are more or less consumptive patients in Sicily or Madeira, or in the West Indies, for the purpose of forming an opinion of the propriety of sending patients there. I would rather inquire, do patients more frequently recover from this disease in any certain climate? Now there can be nothing more evident than that consumption, whatever its relative frequency may be in regard to this country, is very fatal in Madeira. Dr. Gourlay bears testimony to this in strong and decisive terms, by stating that whole families in this island are swept away by it. In Sicily also, such is the dread of the disease, that a family in which it happens is wholly shut out from society, and the least thing is not allowed to be received by a family in health from one in which consumption exists, under a notion that this disorder is contagious. It may therefore be safely inferred, that the disease often affects several of a family, and likewise that consumption must be much dreaded in Sicily on account of its serious nature.

Within the tropics also, where Dr. Buxton has conducted the reader of the Monthly Magazine, if he had found the most complete evidence of the non-existence of the disease, he has only proved that there are circumstances connected with these regions which do not encourage consumption, and therefore the experiment of sending such as may be disposed to it, may be inferred to be fair and allowable; but, while he has not proved, in case of the disease being actually formed, that it is more tractable and less fatal, this information is of no value in the actual cure of consumption. The proposition that the frequency of occurrence of any serious disease shall be commensurate with its fatality, does not in many respects hold good. We may instance that the lock jaw is a very frequent and fatal disease in the West

Indies;

Indies; it is a much less frequent but equally fatal disease in this country.

Another instance of error in which I conceive those recommending equable and high temperature in consumption have fallen, is, in considering an occasional cause, which concurs with others to promote the disease, to be sufficient to point out a remedy for it when formed, and hence show that an opposite condition to that occasional cause should be adopted in the treatment of the disorder. In many instances, however, we have relinquished such a line of proceeding with the most evidently beneficial effects. For example, in many cases of fever in which cold has been evidently a concurring occasional cause; and in typhus, in which cold has as much the effect of promoting its prevalence in winter, in this country, as it contributes to cause consumption—nevertheless, in the treatment, we combine, in connection with other means, the use of cold to alleviate the symptoms, and cure these fevers, although we are aware that severe weather performs a material part in causing them, and rendering them more frequent in certain seasons of the year.

I cannot allow a remark to pass unnoticed, pointing out a sort of refutation of an opinion that I have given. I refer to my intimation, that a certain humidity of atmosphere is desirable to the consumptive, and that an actually contrary state is pernicious, and contributes in some degree to cause the disease. I shall make use of the materials on this subject allowed by the reviewer to be just, and I find that butchers, Scotch fish-wives, catgut makers, stable grooms, and dragoons, are noted to be particularly exempt from consumption in this country. Now all these are much in a comparatively moist atmosphere, and it may be right to remark, that the cure of consumption in a cow-house might to a certain extent be due, if at all preferable, to the humid vapour from the animals below. In addition to this, a remarkable fact is mentioned by Dr. Watts in his book on Hooping Cough, that a set of fishermen in Scotland, who, while at home for some part of the year, and living in idleness within their own habitations, frequently contract severe coughs, which they with certainty lose after having embarked for some little time in their employ on the ocean. In my letters on the efficacy of high and equable temperature for the cure of consumption, other reasons are given in support of this opinion. While conversing with Dr. Gladstone on the subject of the contents of the above-mentioned pamphlet, he related some circumstances respecting consumption which I considered to be very interesting. I therefore requested him to favour me with some of the particulars, which he has obligingly done in the following letter. Dr. Gladstone, at the time alluded to below, was in the capacity of a surgeon of a man-of-war.

"On leaving England for the Mediterranean station, in the year 1804, several men under consumptive complaints were embarked for the benefit of that climate, and others, having a tendency to these complaints, were retained on-board for the same reason. On our arrival, it was found that the experienced medical officers of the fleet on that sta-station, were sending their consumptive patients to a northern climate, as the only chance remaining for their recovery. But few of such patients survive the south-east winds which blow very frequently on the shores of the Mediterranean. So well aware are the physicians of those countries of the fatality of this dry wind in consumptive patients, that they carefully guard them from it. Three out of nine of those patients who embarked with me to the Mediterranean, died during one day's dry wind; the other six were sent to the hospital at Malta, to be invalided to a northern climate. During five years' service in the Mediterranean, I have invariably observed that more consumptive patients have died during the dry summer-months than in the winter, and that the hectic fever is always higher, and the wasting greater, during the dry than the wet season. While in these seas, I had a case of an officer in consumption, who was sent to the hospital at Malta to be invalided for a northern climate; but, on finding his ship ordered to Constantinople for the winter, he re-embarked, and perfectly recovered among three months' fogs equal to any in England. This is a fact which happened under my own eyes. It may be necessary to say, that this officer lost his leg in passing the Dardanelles."

This letter contains facts which coincide greatly with circumstances I have stated in my letters on the efficacy of high and equable temperature in consumption, although the observations of each were made in very different climates. They also place in a strong point of view the impropriety of sending consumptive patients to the Mediterranean shores.

I shall close this communication by relating two cases of marked pulmonary affection, or what may be called consumption of the lungs, with their results. These I relate because they can be well authenticated in all their particulars, and are very intimately connected with the present discussion.

A man of the name of Dean, who keeps a green-shop at Deptford, came to me first about the beginning of December 1813. He had several times spit up blood, and now expectorated what he called phlegm. He had a quick pulse, hectic fever, and night sweats, and was much debilitated and emaciated, emaciated. In the course of this month, finding himself getting worse, he consulted me about the propriety of going to his friends at Chiselhurst, to which I could not object. I gave him the following directions in regard to temperature: that he should get out of doors, if possible, every day; that he should not induge in sitting over the fire, but keep himself warm by clothing. In regard to nourishment, that he should take a little animal food every day, if his appetite led him to wish for it, and drink a small quantity of porter, or wine and water. He came to me several times in a tilted cart during the severe weather, and his disease appeared to vary in no considerable degree for two months and upwards. A small abscess formed in the axilla, which was opened by Mr. Waring of St. Mary Cray. He had, twice or three times, a return of spitting of blood. In March 1814, he called on me, after an interval of a month, improved in every respect. He was some time afterwards in an invalid state, though attending to his concerns, but has passed through the present winter with very little inconvenience. I saw him a week ago in apparent good health; he had lost his emaciation. I have every reason to think that he attended strictly to my directions in regard to temperature, food, and medicine.

A gentleman of the faculty, who acts as an assistant to a relation of his in Deptford, has, previously to the time I shall more particularly mention, been in a very bad state of health, with cough, emaciation, pains in the bowels, and considerable debility; quick pulse, and hectic fever. He has the appearance of a scrofulous habit. In the latter end of January 1814, I was desired to see him, and found him expectorating considerable quantities of matter. I recommended him to be very guarded in respect to temperature, and not to suffer himself to indulge over a large fire, or to keep his sitting-room hot, to both of which I found him much inclined. This advice he entirely neglected, and he grew worse. It happened that several days in February were comparatively warm, and the sun shining strongly on the patient's sitting-room, together with a considerable fire, rendered the apartment particularly hot. During this time, which lasted for seven or eight days, he got evidently and rapidly worse; and his strength failed him so considerably, as not at last to allow him to remove into his sitting-room. He took to his bed in despair. I have now to state, that the bed-room the patient occupied had no fire-place, and was so situated as not to admit the rays of the sun into it, and had undergone no effort in airing than in being constantly used. The weather at this time also changed, and the the temperature of the room could not exceed 45 degrees. During this confinement to bed under these circumstances, the patient gradually got better, and the spitting of matter from the lungs became very inconsiderable in about three weeks. He is now in no worse health than before the above-mentioned attack; and I consider his rescue from death to be owing entirely to his being obliged to keep his chamber, and a chamber of such description.

*Croom's Hill;* THOMAS SUTTON, M.D.

*Feb.* 10*th*, 1815.

---

